# Antidepressant stimulation of CDP-diacylglycerol synthesis does not require monoamine reuptake inhibition

**DOI:** 10.1186/1471-2202-11-10

**Published:** 2010-01-27

**Authors:** Marwa A Aboukhatwa, Ashiwel S Undieh

**Affiliations:** 1 Laboratory of Integrative Neuropharmacology, Department of Pharmaceutical Sciences, Thomas Jefferson University School of Pharmacy, Philadelphia, Pennsylvania, USA; 2 Department of Pharmaceutical Sciences, University of Maryland, Baltimore, Maryland, USA

## Abstract

**Background:**

Recent studies demonstrate that diverse antidepressant agents increase the cellular production of the nucleolipid CDP-diacylglycerol and its synthetic derivative, phosphatidylinositol, in depression-relevant brain regions. Pharmacological blockade of downstream phosphatidylinositide signaling disrupted the behavioral antidepressant effects in rats. However, the nucleolipid responses were resistant to inhibition by serotonin receptor antagonists, even though antidepressant-facilitated inositol phosphate accumulation was blocked. Could the neurochemical effects be additional to the known effects of the drugs on monoamine transmitter transporters? To examine this question, we tested selected agents in serotonin-depleted brain tissues, in PC12 cells devoid of serotonin transporters, and on the enzymatic activity of brain CDP-diacylglycerol synthase - the enzyme that catalyzes the physiological synthesis of CDP-diacylglycerol.

**Results:**

Imipramine, paroxetine, and maprotiline concentration-dependently increased the levels of CDP-diacylglycerol and phosphatidylinositides in PC12 cells. Rat forebrain tissues depleted of serotonin by pretreatment with *p*-chlorophenylalanine showed responses to imipramine or maprotiline that were comparable to respective responses from saline-injected controls. With fluoxetine, nucleolipid responses in the serotonin-depleted cortex or hippocampus were significantly reduced, but not abolished. Each drug significantly increased the enzymatic activity of CDP-diacylglycerol synthase following incubations with cortical or hippocampal brain tissues.

**Conclusion:**

Antidepressants probably induce the activity of CDP-diacylglycerol synthase leading to increased production of CDP-diacylglycerol and facilitation of downstream phosphatidylinositol synthesis. Phosphatidylinositol-dependent signaling cascades exert diverse salutary effects in neural cells, including facilitation of BDNF signaling and neurogenesis. Hence, the present findings should strengthen the notion that modulation of brain phosphatidylinositide signaling probably contributes to the molecular mechanism of diverse antidepressant medications.

## Background

Neither the pathophysiology of depression nor the mechanism of action of various antidepressant agents is fully understood. Accumulating evidence implicates brain phospholipid metabolism in the actions of diverse antidepressant drugs [1-3]. For example, diverse antidepressant agents increase the cellular production of CDP-diacylglycerol and its synthetic derivative, phosphatidylinositol, in depression-relevant regions of the rat brain [2]. Moreover, blockade of downstream inositol phospholipid signaling results in significant disruption of behavioral antidepressant effects in the rat forced swim model of depression [3]. These and related observations have reawakened interest in neural phospholipid systems as potentially crucial contributors to the pathophysiology of depression and/or the mechanism of action of antidepressant drugs.

CDP-diacylglycerol is a crucial intermediate in the synthesis of phosphatidylinositol and related signaling mediators. Enhanced production of CDP-diacylglycerol can be expected to lead to increased synthesis of phosphatidylinositides. Hence, blood platelets incubated with different antidepressants show higher levels of phosphatidylinositides compared to control platelets, and this newly synthesized pool of phosphatidylinositides could be further available for receptor-coupled cell signaling [4,5]. Another recent study also showed that different antidepressant drugs induce phosphatidylinositide synthesis and facilitate subsequent serotonergic-stimulated accumulation of inositol phosphate second messengers [3]. This report also showed that selective blockade of phosphoinositide-linked 5HT_2 _serotonin receptors inhibited the effects of antidepressant drugs on inositol phosphate accumulation, but the drug effects on CDP-diacylglycerol production or phosphoinositide synthesis were not substantially reduced by 5HT_2 _antagonist treatment. These observations suggest that the drug effects on CDP-diacylglycerol and phosphatidylinositide synthesis involve a mechanism that may not depend on increased synaptic serotonin action. Clarifying such a mechanism would increase understanding of depression pathology, and could lead to the development of new and better treatment strategies.

The present study was directed at testing the hypothesis that the nucleolipid effects of antidepressants on CDP-diacylglycerol and its derived inositol phospholipids are substantially independent of the known effects of the drugs to enhance synaptic serotonin levels. We evaluated the neurolipid effects of a selection of drugs in tissues depleted of serotonin, and in neuron-like PC12 cells that are deficient in receptors and transporters for serotonin or norepinephrine [6]. Upon observing that the drugs retained their ability to acutely increase nucleolipid synthesis, we explored if the drugs might directly enhance the enzymatic activity of CDP-diacylglycerol synthase, the enzyme that synthesizes CDP-diacylglycerol. The findings suggest that various antidepressant agents are capable of stimulating the activity of CDP-diacylglycerol, which might explain the enhancing effects of the agents on CDP-diacylglycerol synthesis and phosphatidylinositide production.

## Methods

### Animals

Male inbred C57BL/6 mice weighing 25-30 g were purchased from Harlan (Indianapolis, Indiana) and housed 5 per cage. Male Sprague-Dawley rats weighing 225-250 g were purchased from Zivic Laboratories (Pittsburgh, PA) and housed three per cage. Animals were kept for at least three days before use and housing was on a 12-hour light/dark cycle in climate-controlled facilities. Food and water were freely accessible to each cage of animals. Protocols for the care and use of the animals were approved by the Institutional Animal Care and Use Committee and conformed to the National Institutes of Health Guide for the Care and Use of Laboratory Animals.

### Drugs and chemicals

Buffer reagents and all drugs used were purchased from Sigma-Aldrich (St. Louis, MO). For the neurolipid (CDP-diacylglycerol and phosphatidylinositide) assays and the enzyme activity experiments, test drugs were dissolved in HEPES bicarbonate (HB) buffer, while for the cell culture experiments the drugs were dissolved in phosphate buffered saline. Drugs were prepared fresh before use.

### *p*-Chlorophenylalanine induction of brain serotonin depletion

To deplete rat brain tissues of serotonin content, the animals were administered the tryptophan hydroxylase inhibitor, p-chlorophenylalanine (PCPA) at the dosage of 150 mg/kg daily for three days, followed by a 12-h washout period prior to testing. Control animals received injections of saline. This regimen of PCPA is known to result in depletion of 85-95% of forebrain serotonin content without affecting the catecholamines [7-9].

### Measurement of CDP-diacylglycerol synthesis in serotonin-depleted brain tissues

CDP-diacylglycerol levels in brain tissue preparations were measured as previously described [2,10]. For the present study, hippocampal or frontal cortical tissues were prepared from saline-injected (control) and PCPA-pretreated rats and processed in parallel. Following a 30-min prelabeling incubation with 0.6 μM 5- [^3^H]cytidine (20 Ci/mmole, American Radiolabeled Chemicals, St. Louis, MO), aliquots of slices from each pretreatment group were incubated with various concentrations of selected antidepressant agents for 90 min, and accumulated [^3^H]CDP-diacylglycerol measured as previously described [10].

### Measurement of CDP-diacylglycerol and phosphatidylinositide synthesis in PC12 cells

PC12 cells obtained from ATCC (Manassas, VA) were maintained in RPMI-1640 Medium supplemented with 5% fetal bovine serum, 10% horse serum and 2 mM L-glutamine at 37°C with 5% CO_2 _aeration. The cells were cultured on poly-D-lysine coated plates until reaching approximately 80% confluency and then transferred to Neurobasal medium (Neurobasal+N2 supplement +glutamine) one day before the assay. Cells in wells of a 24-well plate were labeled with 1.5 μCi [5-^3^H]cytidine (20 Ci/mmol; ARC, St. Louis, MO) for 30 min to generate a pool of radiolabeled cytidine triphosphate (CTP). After addition of 5 mM LiCl, solutions of test drugs were added to the cells at indicated concentrations and incubation continued for 3 h. To terminate the reaction, 1.5 ml of chloroform-methanol-1 M HCl (100:200:1) was added with mixing. The lipids were extracted by partitioning to the chloroform layer as described [10,11]; aliquots thereof were quantitatively transferred to polypropylene tubes and dried overnight at room temperature. Biosafe scintillation cocktail was added to each sample and the radioactivity determined by liquid scintillation. The radioactivity in each sample corresponds to [^3^H]CDP-diacylglycerol as characterized in previous studies [10,12,13].

For the assay of phosphatidylinositol synthesis, PC12 cells cultured as described above were labeled for one hour with 1.5 μCi [^3^H]inositol (20 Ci/mmol; ARC, St. Louis, MO). Subsequent drug treatments were maintained for an incubation period of 3 h, followed by extraction and quantitation of the inositol phospholipids as described [10,11].

### Measurement of CDP-diacylglycerol synthase activity

CDP-diacylglycerol synthase activity was assayed in mouse brain membrane preparations according to previously described methods with minor modifications [14-16]. Mice were rapidly decapitated and the desired tissues dissected, chopped into 350-μm slices, and washed thrice with calcium-free HB buffer. Aliquots of tissue slices corresponding to approximately 500 μg protein were distributed into tissue culture wells containing 450 μl HB buffer and incubated at 37°C. Various concentrations of each test drug in 50 μl volumes were added to appropriate tubes and incubation continued for 3 h. Afterwards, the tissues were transferred into cold Tris lysis buffer (pH 7.8) containing a cocktail of protease inhibitors (Sigma-Aldrich, St. Louis, MO) and lysed by gentle homogenization. Cell debris was removed by centrifugation (550 × g, 10 min, at 4°C) and protein concentration of the membrane preparations was determined by the Bradford method. Equal aliquots of 25 μg membrane protein were preincubated for 25 min with 3 μM 5- [^3^H]CTP (specific activity 13.9 Ci/mmol; Moravek Biochemicals, Brea, CA) in a 50-μl reaction mixture that contained 0.1 M Tris-HCl pH 7.5, 0.2 M KCl, 1 mg/ml bovine serum albumin, 0.1% Triton X-100, 0.25 mM dithiothreitol, and 200 μM 1,2-dioleoyl-sn-glycero-3-phosphate. The reaction was started by the addition of 10 mM MgCl_2 _and stopped after 20 min by the addition of chloroform: methanol: 10 M HCl (1:2:0.02, v/v). After incubating at room temperature for 30 min to extract formed [^3^H]CDP-diacylglycerol, the amount of the incorporated radioactivity was determined by liquid scintillation counting. Assayed enzyme activity was linear with time up to at least 30 min and with protein concentration up to at least 50 μg. One unit of CDP-diacylglycerol synthase activity was defined as the amount of the enzyme that catalyzed the formation of one nanomole of CDP-diacylglycerol per minute.

### Data analysis

Neurolipid data were calculated as dpm/mg protein. Each set of data was normalized by taking percentages relative to control incubations that excluded drug treatments. Data from two or three separate runs of the experiments were then pooled for statistical analysis and graphical representation as shown. Similarly, enzyme activity data were normalized against each day and tissue controls, and the data pooled for analysis. Statistical analyses were by One-Way ANOVA followed with posthoc Tukey tests to compare all possible pairs of group means or the Dunnett test to compare each treatment mean to its respective control group as indicated in the figure legends. Statistical comparisons were considered significant at p < 0.05 or better.

## Results

### Effects of serotonin depletion on the effectiveness of various antidepressant agents

**Figure 1:**
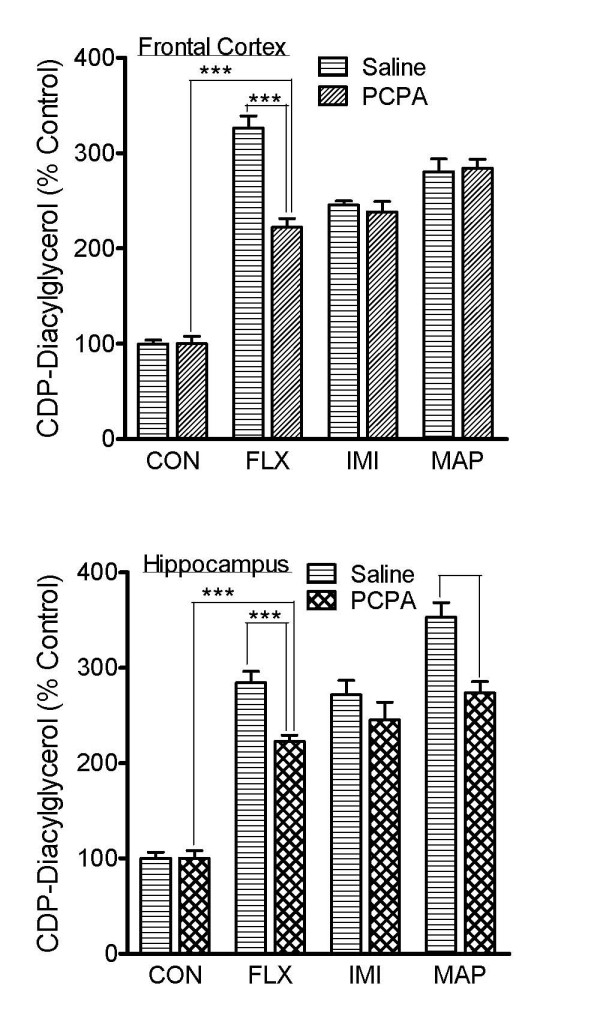
**Effects of PCPA-induced serotonin depletion on antidepressant stimulation of CDP-diacylglycerol synthesis in brain tissue**. Brain slices were prepared from the frontal cortex or hippocampus tissues of saline-injected control rats or animals that had been administered *p*-chlorophenylalanine (PCPA) to deplete endogenous serotonin stores. The tissue slices were labeled with [^3^H]cytidine and then incubated with predetermined concentrations of the antidepressant drugs fluoxetine (FLX, 100 μM), imipramine (IMI, 300 μM), or maprotiline (MAP, 100 μM). After 90 min, accumulated [^3^H]CDP-diacylglycerol was measured. Data were calculated as percentages relative to the respective basal accumulations in the control or PCPA group. Each bar represents the mean ± SEM (N = 6). PCPA treatment had no significant effect on basal [^3^H]CDP-diacylglycerol accumulation in either tissue; such basal effects being, respectively, 8194+806 v. 8555+1657 for control versus PCPA in frontal cortex, and 8026+1295 v. 8139+1642 for control versus PCPA in hippocampus. Each drug induced significant accumulations of [^3^H]CDP-diacylglycerol in either the control or PCPA tissues (ANOVA, p < 0.0001 for each drug). PCPA partially reduced FLX and MAP effects but did not eliminate any of the drug effects: ***p < 0.001, compared by posthoc Tukey tests.

Control and serotonin-depleted tissues from the rat hippocampus or frontal cortex were tested for CDP-diacylglycerol responses to various antidepressant agents. The tissue slices were prelabeled with [^3^H]cytidine and then incubated with maximally effective concentrations of the antidepressant drugs fluoxetine (FLX, 100 μM), imipramine (IMI, 300 μM), or maprotiline (MAP, 100 μM); these dose selections were based on previously published studies with brain slice preparations [2,3]. While similar results were obtained in the hippocampus and frontal cortex, the results for the frontal cortex are shown in Fig. 1. Imipramine, fluoxetine, or maprotiline induced significant accumulations of CDP-diacylglycerol in the saline-pretreated control tissues as expected. Tissues depleted of serotonin also gave significant responses to the antidepressant agents, although the responses to fluoxetine in the frontal cortex and to fluoxetine and maprotiline in the hippocampus were significantly but partially (21-32%) reduced in *p*-chlorophenylalanine (PCPA)-pretreated tissues. The concentration and conditions of PCPA treatment in these experiments are known to result in practically complete depletion of serotonin from the tissues [7-9].

### Antidepressant drugs increase the synthesis of CDP-diacylglycerol and phosphatidylinositides in PC12 cells

**Figure 2:**
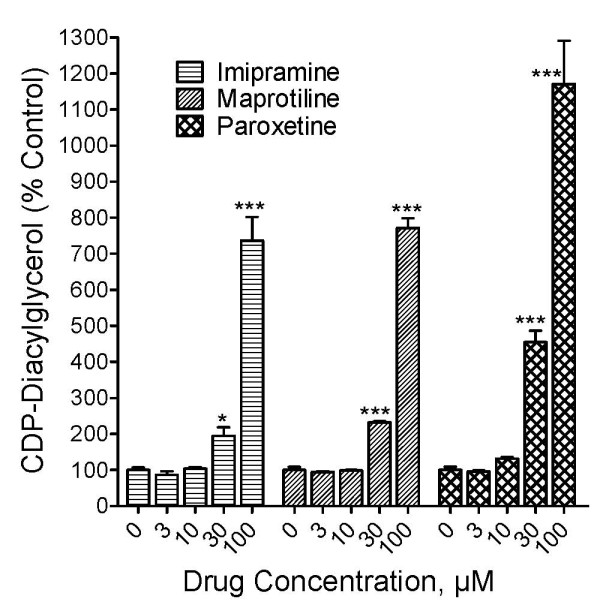
**Effects of antidepressant agents on [^3^H]CDP-diacylglycerol synthesis in PC12 cells**. PC12 cells were prelabeled with [^3^H]cytidine for 30 min and cells in different culture wells incubated with various concentrations of the antidepressant drugs imipramine, paroxetine or maprotiline. [^3^H]CDP-diacylglycerol was determined after 3 h. Data were calculated as percentages relative to the control incubations that received no drug treatment. Each bar represents the mean ± SEM (N = 3 experiments). The drug treatments increased [^3^H]CDP-diacylglycerol significantly (ANOVA, p < 0.0001 for each drug). *Posthoc *Dunnett tests showed statistically significant increases in CDP-diacylglycerol at 30 and 100 μM. *p < 0.05; ***p < 0.001, compared to controls.

PC12 cells prelabeled with [^3^H]cytidine were incubated with different concentrations of antidepressant drugs ranging from 0.1 μM to 300 μM and the accumulation of CDP-diacylglycerol assayed after 3 h. The results are shown in Fig. 2. Imipramine (tricyclic antidepressant), maprotiline (norepinephrine and serotonin reuptake inhibitor), and paroxetine (selective serotonin reuptake inhibitor) each significantly and concentration-dependently increased the levels of CDP-diacylglycerol in the PC12 cells [ANOVA, p < 0.0001]. Based on the *posthoc *Dunnett tests, significant effects were obtained at concentrations of 30 μM and higher.

**Figure 3:**
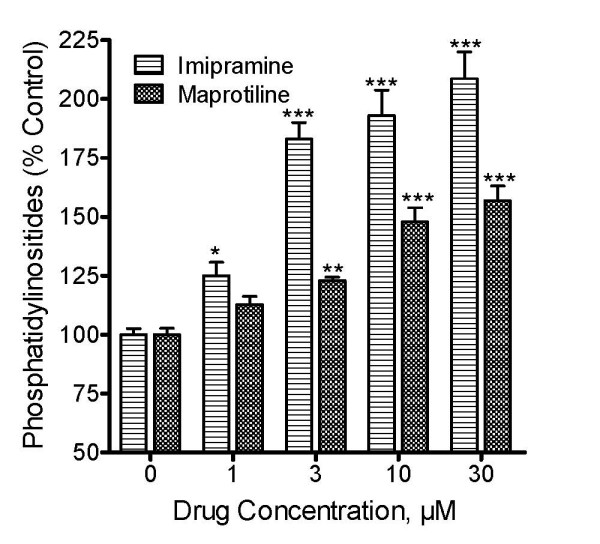
**Effects of antidepressant agents on phosphatidylinositide synthesis in PC12 cells**. PC12 cells were labeled with [^3^H]inositol and cells in different culture wells incubated with various concentrations of selected antidepressant drugs. After 3 h, accumulated [^3^H]phosphatidylinositides were determined and the data calculated as percentages relative to control. Each bar represents the mean ± SEM (N = 3 experiments). Each drug treatment increased the levels of [^3^H]phosphatidylinositides significantly and concentration-dependently (ANOVA, P < 0.0001 for each drug). *Posthoc *Dunnett tests showed statistically significant increases in phosphatidylinositides at 1-30 μM for imipramine and at 3-30 μM for maprotiline. *p < 0.05; ** P < 0.01; ***p < 0.001; compared to controls.

To test if antidepressant-induced increases in CDP-diacylglycerol translated into enhanced synthesis of phosphatidylinositides in PC12 cells, another batch of the cells was prelabeled with [^3^H]inositol and aliquots tested with the drugs. Maprotiline and imipramine significantly and dose-dependently increased phosphatidylinositide levels in PC12 cells (Fig. 3). The effects of imipramine were statistically significant at concentrations of 1 μM and higher, while the effects of maprotiline became significant at 3 μM and higher concentrations. Ten micromolar and higher concentrations of fluoxetine and paroxetine were also tested for effects on phosphatidylinositides; while the 10 and 30 μM concentrations showed significant enhancement of phosphatidylinositide synthesis, the 100 μM and higher concentrations caused a reduction of labeling below control levels, suggesting possible toxicity at the higher concentrations (data not shown). Taken together, the two neurolipid results (Figs. 2 and 3) suggest that antidepressants can increase CDP-diacylglycerol synthesis which translates to increased phosphatidylinositide synthesis in PC12 cells.

### Antidepressants increase CDP-diacylglycerol synthase activity in mouse frontal cortex

**Figure 4:**
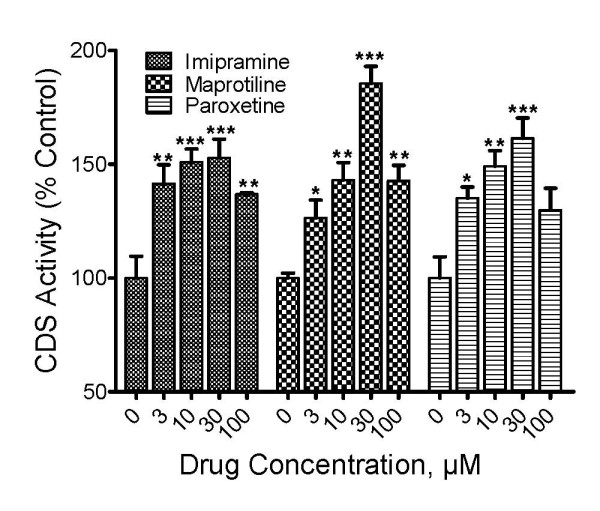
**Antidepressant drug effects on CDP-diacylglycerol synthase activity in mouse cortex tissues**. The enzymatic activity of CDP-diacylglycerol synthase (CDS) was measured in mouse cortical tissue preparations by determining the incorporation of radiolabeled cytidine derived from 5- [^3^H]CTP into CDP-diacylglycerol. Antidepressant agents were tested at concentrations ranging from 1 μM to 100 μM. Data from each assay were normalized against the control samples by calculating the percentages shown. Each bar is the mean ± SEM (N = 3 experiments). One-Way ANOVA analyses for each drug showed significant effects for imipramine (p < 0.001), maprotiline (p < 0.0001), and paroxetine (p < 0.01). *Posthoc *Dunnett tests showed that imipramine and maprotiline elicited significant effects at 3-100 μM concentrations while with paroxetine significant effects were evident at concentrations of 3-30 μM. *p < 0.05; ** P < 0.01; ***p < 0.001, compared to controls.

The enzyme activity of CDP-diacylglycerol synthase (CDS) was determined by measuring the rate of incorporation of tritiated CTP into CDP-diacylglycerol, and the results are shown in Fig. 4. Each of the antidepressants, imipramine, paroxetine, and maprotiline, significantly increased CDS activity (ANOVA, p < 0.001 for each drug). The *posthoc *Dunnett tests showed that the effects of each drug attained statistical significance at concentrations as low as 3 μM, and then peaked right afterwards at 10 μM (imipramine) or 30 μM (paroxetine and maprotiline). Thus, drug stimulation of CDS activity may underlie the positive drug effects on CDP-diacylglycerol synthesis.

## Discussion

The classic action of antidepressants is to inhibit the reuptake of monoamines which leads to increased synaptic levels of the transmitters. The subsequent postsynaptic actions of the monoamines on their cognate signaling systems may then lead on to produce the clinical antidepressant effect [17-19]. However, such postsynaptic actions have not been fully elucidated, although various tertiary signaling effects ranging from alterations of ion channel activity to modulation of protein kinases and gene transcription have been proposed [17,20-22]. Taking clues from a recent study showing that antidepressants concentration-dependently increase the levels of CDP-diacylglycerol and phosphatidylinositides in brain tissue preparations [2], we have now addressed the question of the extent to which this neurolipid effect may involve actions of the monoamine transmitters. The data demonstrate that various antidepressants increase phosphatidylinositol and CDP-diacylglycerol synthesis probably through stimulation of the enzymatic activity of CDS, the enzyme that synthesizes CDP-diacylglycerol. While not all antidepressant agents depend on serotonin signaling for their actions [9], a demonstration that the neurolipid effects of selective serotonin reuptake inhibitors involves multiple mechanisms should suffice as proof of the concept that not all actions of antidepressants depend on monoamine reuptake inhibition.

PCPA is often used to deplete tissues of serotonin content either in vivo or in vitro [7-9]. Protocols used here were similar to those used in numerous other studies some of which had the residual content of serotonin assayed to assure the expected depletion. Thus, even though we did not measure residual serotonin in the present study, there is strong precedent to assume that the agent worked as expected. Moreover, the significant reduction of the effect of fluoxetine (a selective serotonin reuptake inhibitor) in the PCPA-treated tissues supports the presumption that there was depletion of the monoamine. Unlike the fluoxetine results, there was no inhibition of imipramine (a serotonin/norepinephrine reuptake inhibitor) in any tissue, or of maprotiline (a norepinephrine reuptake inhibitor) in the frontal cortex, thus indicating that the actions of these agents on CDP-diacylglycerol or phosphatidylinositide synthesis do not require serotonin. A desirable complementary experiment would be to test if other tissues that are depleted of norepinephrine would show reductions in imipramine or maprotiline responses.

PC12 cells are widely used as a model of neuronal cell metabolism [23,24]. The primary reason we chose PC12 cells was to use a model system that represents practically complete lack of endogenous monoamines, notably serotonin and norepinephrine [6]. The marked and dose-dependent stimulation of CDP-diacylglycerol and phosphatidylinositide synthesis by imipramine as well as maprotiline and paroxetine in PC12 cells implies that monoamine neurotransmitters do not play a major role in the nucleolipid effects of the drugs. Interestingly, there was no significant effect of the drugs on inositol phosphate accumulation in PC12 cells (data not shown). The accumulation of inositol phosphates requires receptor-coupled stimulation of phospholipase C. Hence, while the antidepressants are able to enhance the production of substrate for the phospholipase C reaction, the drugs are apparently incapable by themselves to induce the breakdown of these substrates into active second messengers. This observation is in agreement with our previous proposition of a tandem signaling model for antidepressant-monoamine transmitter function. By this model, the antidepressants increase the availability of phosphatidylinositide substrates and then enhance the synaptic levels of the monoamines which then act on their cognate postsynaptic receptors to convert the phosphatidylinositide substrates into functional second messengers.

It was noted that the antidepressant drugs significantly enhanced phosphatidylinositide levels at concentrations that were clearly lower than the concentrations necessary to observe significant accumulations of CDP-diacylglycerol. Given that CDP-diacylglycerol synthase activity is considered to be rate-limiting, it is likely that any CDP-diacylglycerol formed is quickly converted to phosphatidylinositide. As such, accumulation of CDP-diacylglycerol may not become evident until the mechanism for synthesizing phosphatidylinositides begins to saturate at the higher drug concentrations. Additionally, phosphatidylinositol is able to accumulate because, as explained above, there is no mechanism to stimulate the activity of phospholipase C which is required to break down the phospholipid. Based on the tandem signaling model, one may predict that in the in vivo environment the direct CDP-diacylglycerol effect and the indirect monoamine reuptake effects of the antidepressant drugs would work in concert to enhance both the generation of substrate and the receptor-mediated release of intracellular second messengers.

Following the foregoing results, it still remained to be investigated how the antidepressants increase the levels of the neurophospholipids in a direct manner that may not be dependant on monoamine reuptake inhibition. First, we considered if this action was related to the lipidosis that has been associated with a number of amphiphilic compounds, a group of chemical structures that includes the tricyclics and several other antidepressants. Amphiphiles are thought to induce lipidosis (nonspecifically increased lipid synthesis) through inhibition of phospholipases that then progresses to the inhibition of phospholipid degradation [25-27]. Other observations suggest that increased cellular phosphatidylinositide levels have an inductive effect that increases non-phosphatidylinositide-derived glycerolipid synthesis [28]. If so, then a primary action of antidepressants on CDP-diacylglycerol synthesis could lead to increased phosphatidylinositide synthesis, which may then enhance the synthesis of other non-phosphatidylinositide-containing lipids.

It is still unclear if imipramine or any of the other agents interact directly with the phospholipids to prevent their degradation or inhibit phospholipases [29]. If the tested antidepressant drugs induced phosphatidylinositide accumulation solely through inhibition of phosphatidylinositide metabolism, then there should not have been an increase in CDP-diacylglycerol, which is upstream of phosphatidylinositol and whose levels are tightly controlled by the rate-limiting activity of CDS. Moreover, in brain slices, co-incubation of antidepressant with direct agonists of 5HT_2 _receptors resulted in enhanced accumulations of inositol phosphates over and above the effects due to agonist alone, implying that phosholipase C may not have been inhibited (or at least that enzyme stimulation by a coupled receptor was not prevented) [3]. Taken together, it appears that enhanced synthesis rather than inhibited degradation may explain the observed drug-induced increases in the levels of signaling phospholipids.

Based on the observation that chemically diverse antidepressants increased the synthesis of the phospholipids, we hypothesized that the drugs enhance the activity of the phospholipid synthesizing enzymes. This prompted the need to investigate the effects of the drugs on CDP-diacylglycerol synthase activity. CDS is reported to have high activity in the brain based on distribution analysis in rodent tissues [30]. Further, the cortex was selected for testing seeing that cognitive abnormalities such as hopelessness, memory impairments, suicidality and guilt in many depressed patients are thought to be associated with, or mediated through, the frontal cortex region of the brain. In general, the cortex might also regulate the emotional behavior abnormalities [31]. Although the tested drugs were chemically different, the drugs shared a common enhancing effect on CDS activity in the cortex. CDS activity was increased at drug concentrations of 3 μM and above, which is consistent with our previous report where the antidepressants increased CDP-diacylglycerol accumulation in rat cortex slices at concentrations of 1-3 μM and above [2]. That all three tested agents enhanced the enzymatic activity of CDS provides a plausible explanation for the enhanced synthesis of CDP-diacylglycerol and phosphatidylinositides. The detailed mechanism by which the drugs induce CDS activity, however, remains to be explored.

One concern about these observations was the potential toxicity of the antidepressants given that the effective concentrations were in the micromolar range (3-300 μM). A previous report showed that antidepressants such as fluoxetine (selective serotonin reuptake inhibitor) and amitriptyline (non selective reuptake inhibitor) did not adversely affect cell survival of PC12 cells up to 100 μM for 24 or 48 h incubation periods [32]. These drugs even appeared to exert a neuroprotective effect on PC12 cells against neurotoxic insults like 200 μM hydrogen peroxide by upregulating superoxide dismutase activity [32]. In another recent study examining the anti-inflammatory properties of tricyclic antidepressants, concentrations of 10 or 20 μM imipramine did not reduce cell viability in the MTT assay in microglia and astrocytes incubated in vitro for up to 24 h [33]. In humans undergoing therapy with imipramine, drug levels reach on average 0.54 μM in the plasma [34,35]. Imipramine and similar drugs, however, accumulate in brain tissue 32-fold higher than the level in the plasma or the serum [36]. If so, then the final concentrations to which the brain is exposed are actually comparable to the concentrations of imipramine that showed significant effects in each of the present measures.

## Conclusions

In conclusion, the results of this study raise the possibility that the neurolipid effects of antidepressant agents, particularly on CDP-diacylglycerol and phosphatidylinositide synthesis, may be independent of monoamine transmitter action. Given that resting cellular levels of CDP-diacylglycerol are thought to be rather low, and that CDS activity is the rate - limiting step in CDP-diacylglycerol synthesis, enhancing the activity of CDS appears to be a logical or plausible explanation for the effect of antidepressants on CDP-diacylglycerol and phosphatidylinositide synthesis.

## Authors' contributions

MAA participated in experimental design, conducted the experiments, and drafted the manuscript. ASU designed the study, and participated in data analysis, writing of the manuscript, and securing funds for the study. Both authors read and approved the final manuscript.

## Acknowledgements

This research was supported by grant #R01DA017614 from the US National Institutes of Health.
